# ESC Congress 2023

**DOI:** 10.1016/j.eclinm.2023.102214

**Published:** 2023-09-01

**Authors:** Charlotte Rowbottom

## Updates to ESC guidelines

The opening session of European Society of Cardiology (ESC) Congress 2023 offered an introduction to the several updates to ESC guidelines premiering at the conference. These updates include recommendations (1) on the management of endocarditis; (2) to reduce cardiovascular risk in patients with diabetes; (3) on treatment of acute coronary syndromes; and (4) on the treatment of cardiomyopathies—the first international guidelines on this topic.

Notable updates include a new diagnostic algorithm for endocarditis; the introduction of a new scoring system, SCORE2-Diabetes, to estimate the 10-year risk of fatal and non-fatal myocardial infarction and stroke in patients with type 2 diabetes; a new section on the management of acute coronary syndromes in patients with cancer and the combining of the 2017 and 2020 guidelines for patients with and without ST-segment elevation.

An interim update on heart failure—the focus of this year's congress—was also presented, reflecting the huge progress in this field since ESC's 2021 guidelines for the diagnosis and treatment of acute and chronic heart failure. More than 10 randomised controlled trials have been released within this time. New recommendations are provided in three areas: chronic heart failure, acute heart failure, and comorbidities and prevention. Integral to ESC guidelines are the scoring tables used to indicate whether a therapy is recommended or not (Class 1–3) and the level of confidence in the recommendations (level A–C), where Class 1A is the highest. This interim update only included results that would lead to new or changed Class 1 or 2A recommendations and at least 75% of taskforce members had to agree on inclusions. Notable updates include a historic Class 1A recommendation for use of a sodium–glucose co-transporter 2 (SGLT2) inhibitor in heart failure with preserved and mildly reduced ejection fraction (HFpEF and HPmrEF).

The inclusion of patient perspectives in these guideline updates is welcome; with the inclusion of new sections on patient-centred care stressing the importance of respecting individual preferences, explaining alternatives, and including patients in decision making as much as possible.

## Inaugural guidelines on cardiomyopathies

Presenting the inaugural guidelines of cardiomyopathies, Elena Arbelo (University of Barcelona, Barcelona, Spain) stressed the importance of the systematic evaluation process to arrive at diagnosis and guide subsequent management, and of a multidisciplinary approach to care. A shared and coordinated care approach between cardiomyopathy specialists and general adult and paediatric cardiology centres is strongly recommended (Class 1C). Two Class 1C recommendations are given for exercise, presented by Juan Pablo Kaski (University College London, London, UK): an individualised risk assessment for exercise for all patients and regular low-to-moderate intensity exercise in all able individuals. At this stage, recommendations are mostly based on observational studies and expert opinion (ie, level C).

## STEP HFpEF

The results from the STEP-HFpEF trial were presented by Mikhail Kosiborod (Saint Luke's Mid America Heart Institute, Kansas City, MO, USA) during the opening Hot Line session on day 1. In the multicentre trial (96 sites across 13 countries), 529 patients with HFpEF and a body-mass index of at least 30 mg/kg^2^ were randomly assigned (1:1) to receive once-weekly semaglutide (2.4 mg) or placebo for 52 weeks. The two primary outcomes were change from baseline in the Kansas City Cardiomyopathy Questionnaire clinical summary score (KCCQ-CSS) and mean percentage change in bodyweight. Secondary outcomes included physical function (via mean change in 6-min walk distance) and inflammatory markers. Treatment with semaglutide led to greater improvements in KCCQ-CSS (16.6 points with semaglutide *vs* 8.7 points with placebo [95% 4.8 to 10.9]; p<0.001) and exercise function (21.5 m with semaglutide *vs* 1.2 m with placebo [8.6 to 32.1]; p<0.001), and greater weight loss (−13.3% with semaglutide *vs* −2.6% with placebo [−11.9 to −9.4]; p<0.001) than placebo. Inflammatory markers were also significantly reduced with semaglutide versus placebo. Serious adverse events were significantly more common in the placebo group (13.3% *vs* 26.7% for semaglutide; p<0.001). The weight loss findings are unsurprising, given what is known about semaglutide, but the improvements in heart failure-related symptoms, inflammatory markers, and physical function represent big potential for clinical benefit in patients with HFpEF.

## QUEST

For the first time, a randomised controlled trial has shown a traditional Chinese medicine to be safe and more effective than placebo, plus standard care, for chronic heart failure therapy. In the late breaking, double-blind QUEST trial, conducted in 133 hospitals across China and Hong Kong, 3100 adults with HFrEF (left ventricular ejection fraction ≤40% and NT-proBNP ≥450 pg/mL) were randomly assigned (1:1) to receive either qiliqiangxin (four capsules, three times daily) or placebo, in addition to standard medications for chronic heart failure. Qiliqiangxin is a traditional Chinese medicine extract obtained from 11 types of herbs, including milkvetch root and gingseng. With qiliqiangxin, the primary outcome—a composite of readmission to hospital for worsening heart failure or cardiovascular death—was significantly less common than in the placebo group (25.02% [n=389] *vs* 30.03% [n=467]; hazard ratio 0.78 [95% CI 0.68 to 0.90]; p<0.001). Qiliqiangxin was well tolerated, with no substantial differences between the two study groups in terms of adverse events or all-cause death. The authors, represented by Xinli Li (The First Affiliated Hospital of Nanjing Medical University, Nanjing, China) during the Hot Line 2 presentation, conclude that these findings demonstrate meaningful clinical benefit and support its use as an adjunct therapy.

## ECLS-SHOCK

In this multicentre, randomised controlled ECLS-SHOCK trial, 420 patients with acute myocardial infarction and cardiogenic shock received either early revascularisation extracorporeal life support (ECLS group) plus standard care or standard care alone (control group). The primary outcome was all-cause mortality at 30 days. The findings were neutral, showing no difference in the primary outcome and, in fact, increased risk of moderate-to-severe bleeding and of peripheral ischaemia (secondary outcomes) in those who received ECLS. Specifically, at 30 days, all-cause mortality had occurred in 100 (47.8%) of 209 patients in the ECLS group and in 102 (49.0%) of 208 patients in the control group (relative risk 0.98 [95% CI 0.80 to 1.19]; p=0.81). These trial findings were presented alongside an individual participant data meta-analysis, published in *The Lancet*, that included all four randomised controlled trials now published on this topic. Presenting, Holger Thiele (University of Leipzig, Leipzig, Germany) concluded that these two papers provide robust evidence that there is no benefit of ECLS in this patient group—thus challenging current guidelines recommendations.

## MULTISTARS-AMI

Presented by Barbara E Stähli (University Hospital Zurich, Zurich, Switzerland) during Hot Line 6, the MULTISTARS AMI open-label, randomised, non-inferiority trial aimed to determine whether immediate complete revascularisation at the time of primary percutaneous coronary intervention (PCI) was non-inferior to staged (19–45 days) multivessel PCI. Eligible participants, from 37 sites in Europe, were haemodynamically stable individuals with ST-segment elevation myocardial infarction (STEMI) and multivessel coronary artery disease. Patients with cardiogenic shock, left main coronary-artery disease, a chronic total occlusion, or previous coronary-artery bypass graft surgery were excluded. Participants were randomly assigned (1:1) to either immediate (n=418) or staged (n=422) PCI. The primary endpoint was a composite of all-cause death, non-fatal myocardial infarction, stroke, unplanned ischaemia-driven revascularisation, or hospitalisation for heart failure within 1 year. At 1 year, 35 patients (8.5%) in the immediate group and 68 patients (16.3%) in the staged group had met the primary endpoint (risk ratio 0.52 [95% CI 0.38 to 0.72]; p<0.001 for non-inferiority). On safety, 104 patients in the immediate group and 145 patients in the staged group had a serious adverse event. Non-fatal myocardial infarction and unplanned ischaemia-driven revascularisation were more common in the staged group than in the immediate group (22 [5.3%] *vs* eight [2.0%] patients and 39 [9.3%] *vs* 17 (4.1%) patients, respectively). All-cause death, stroke, and admission to hospital for heart failure did not differ between the two groups. Summarising the findings and clinical implications, Barbara E Stähli noted “immediate PCI of non-culprit lesions is as effective and safe as a staged procedure.”

## ONCO DVT

Findings from the multicentre, open-label superiority ONCO DVT trial in patients with cancer and distal deep vein thrombosis (DVT) showed that 12 months of edoxaban (an oral factor Xa inhibitor) is superior to 3 months for the reduction of thrombotic events. In ONCO DVT, presented during Hot Line 9 by Yugo Yamashita (Kyoto University, Kyoto, Japan), 604 patients with various types of cancer from 60 sites in Japan were enrolled. After exclusions, 601 patients were included in the intention-to-treat population: 296 in the 12-month group and 305 in the 3-month group. The primary outcome was symptomatic recurrent venous thromboembolism (VTE) or VTE-related death event at 12-month follow-up. A major bleeding event at 12 months was the only secondary outcome. Three patients (1.0%) in the 12-month group and 22 (7.2%) in the 3-month group achieved the primary outcome (odds ratio 0.13 [95% CI 0.03 to 0.44]). The secondary outcome occurred in 28 patients (9.5%) in the 12-month group and 22 (7.2%) in the 3-month group (odds ratio 1.34 [0.75 to 2.41]).

